# Correction: Khayyat et al. Xylitol Inhibits Growth and Blocks Virulence in *Serratia marcescens*. *Microorganisms* 2021, *9*, 1083

**DOI:** 10.3390/microorganisms13102233

**Published:** 2025-09-24

**Authors:** Ahdab N. Khayyat, Wael A. H. Hegazy, Moataz A. Shaldam, Rasha Mosbah, Ahmad J. Almalki, Tarek S. Ibrahim, Maan T. Khayat, El-Sayed Khafagy, Wafaa E. Soliman, Hisham A. Abbas

**Affiliations:** 1 Department of Pharmaceutical Chemistry, Faculty of Pharmacy, King Abdulaziz University, Jeddah 21589, Saudi Arabia; ankhayyat@kau.edu.sa (A.N.K.); ajalmalki@kau.edu.sa (A.J.A.); tmabrahem@kau.edu.sa (T.S.I.); mkhayat@kau.edu.sa (M.T.K.); 2 Department of Microbiology and Immunology, Faculty of Pharmacy, Zagazig University, Zagazig 44519, Egypt; hishamabbas2008@gmail.com; 3 Department of Pharmaceutical Chemistry, Faculty of Pharmacy, Kafrelsheikh University, Kafr El-Sheikh 33516, Egypt; dr_moutaz_986@yahoo.com; 4 Infection Control Unit, Zagazig University Hospitals, Zagazig University, Zagazig 44519, Egypt; rashamosbah6@gmail.com; 5 Faculty of Oral and Dental Medicine, Ahram Canadian University, Giza Governorate 12573, Egypt; 6 Department of Pharmaceutical Organic Chemistry, Faculty of Pharmacy, Zagazig University, Zagazig 44519, Egypt; 7 Department of Pharmaceutics, College of Pharmacy, Prince Sattam Bin Abdulaziz University, Al-Kharj 11942, Saudi Arabia; e.khafagy@psau.edu.sa; 8 Department of Pharmaceutics and Industrial Pharmacy, Faculty of Pharmacy, Suez Canal University, Ismailia 41552, Egypt; 9 Department of Biomedical Science, Faculty of Clinical Pharmacy, King Faisal University, Alhofuf, Al-Ahsa 36362, Saudi Arabia; wafaaezz2006@yahoo.com; 10 Department of Microbiology and Biotechnology, Faculty of Pharmacy, Delta University for Science and Technology, Mansoura 11152, Egypt

In the original publication [1], there was a mistake in Figure 1A as published. The corrected Figure 1 appears below. 

The authors state that the scientific conclusions are unaffected. This correction was approved by the Academic Editor. The original publication has also been updated.

## Figures and Tables

**Figure 1:**
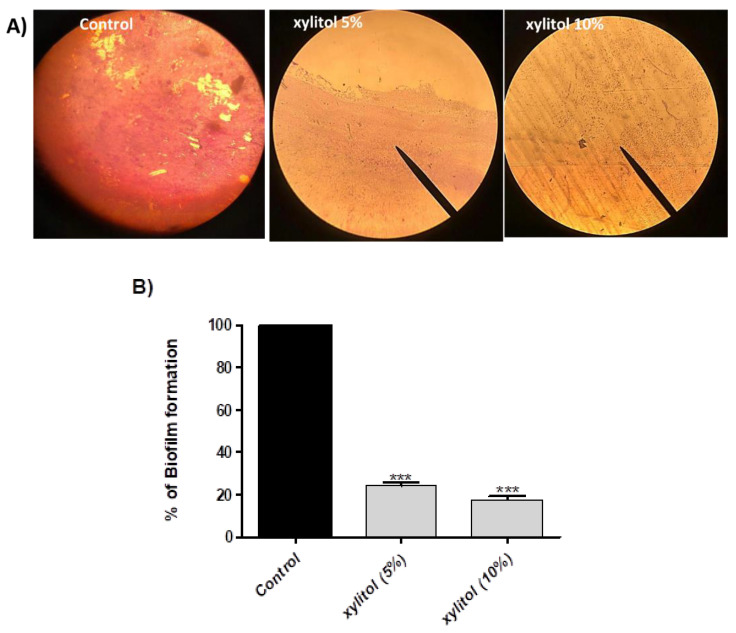
Biofilm inhibition of *S. marcescens* by xylitol. (**A**) Microscopic examination of biofilm inhibition by xylitol. Biofilm was formed on glass cover slips by xylitol-treated and untreated bacteria, and light microscopy was used for visualization. Both thickness and biofilm biomass were reduced by xylitol. (**B**) Biofilm was allowed to form on microtiter plate wells in the presence or absence of xylitol (5% and 10%) and adherent cells were stained with crystal violet, glacial acetic acid was added to dissolve the dye, and the absorbance was measured at 590 nm. The test was performed in triplicate. Biofilm formation was significantly reduced by xylitol. The data shown are the means ± standard errors. One-way ANOVA test followed by Dunnett’s Multiple Comparison test was used for statistical analysis. ***: *p* ≤ 0.001.
